# CCDC86 is a novel Ki-67-interacting protein important for cell division

**DOI:** 10.1242/jcs.260391

**Published:** 2023-01-25

**Authors:** Konstantinos Stamatiou, Aldona Chmielewska, Shinya Ohta, William C. Earnshaw, Paola Vagnarelli

**Affiliations:** ^1^ College of Health, Medicine and Life Sciences, Department of Life Sciences, Brunel University London, London UB8 3PH, UK; ^2^ Wellcome Centre for Cell Biology, Institute of Cell Biology, University of Edinburgh, Edinburgh EH16 4SB, UK; ^3^ Institute for Genetic Medicine, Hokkaido University, Kita-15, Nishi-7, Kita-Ku, Sapporo 060-0815, Japan

**Keywords:** Cell division, Chromosomes, MYCN, CCDC86, Ki-67

## Abstract

The chromosome periphery is a network of proteins and RNAs that coats the outer surface of mitotic chromosomes. Despite the identification of new components, the functions of this complex compartment are poorly characterised. In this study, we identified a novel chromosome periphery-associated protein, CCDC86 (also known as cyclon). Using a combination of RNA interference, microscopy and biochemistry, we studied the functions of CCDC86 in mitosis. CCDC86 depletion resulted in partial disorganisation of the chromosome periphery with alterations in the localisation of Ki-67 (also known as MKI67) and nucleolin (NCL), and the formation of abnormal cytoplasmic aggregates. Furthermore, CCDC86-depleted cells displayed errors in chromosome alignment, altered spindle length and increased apoptosis. These results suggest that, within the chromosome periphery, different subcomplexes that include CCDC86, nucleolin and B23 (nucleophosmin or NPM1) are required for mitotic spindle regulation and correct kinetochore–microtubule attachments, thus contributing to chromosome segregation in mitosis. Moreover, we identified *CCDC86* as a MYCN-regulated gene, the expression levels of which represent a powerful marker for prognostic outcomes in neuroblastoma.

## Teaser

**Highlighted Article:** CCDC86 was identified as a novel component of the perichromosomal layer and was found to be important for chromosome segregation. It represents a strong prognostic marker for neuroblastoma patients.

## INTRODUCTION

As cells enter mitosis, chromatin undergoes a remarkable series of structural changes that lead to chromosome condensation and the formation of individual mitotic chromosomes (Paulson et al., 2021; Booth and Earnshaw, 2017). The composition and function of specific mitotic chromosome domains, such as the chromosome scaffold, centromere or kinetochore, and telomeres, have been extensively investigated. However, the ill-defined compartment known as the chromosome periphery, or perichromosomal sheath or layer, is less characterised. The perichromosomal layer coats the outer surfaces of individual mitotic chromosomes (Dimario, 2004; Hernandez-Verdun and Gautier, 1994; Van Hooser et al., 2005) and constitutes approximately one-third of the protein mass of mitotic chromosomes (Booth et al., 2016; Booth and Earnshaw, 2017). This chromosome compartment appears at prometaphase and disappears at telophase when the nuclear envelope reforms (Montgomery, 1900).

Four different functions have been proposed for the chromosome periphery: (1) involvement in the maintenance of mitotic chromosome structure (Van Hooser et al., 2005), (2) establishment of a physical barrier protecting mitotic chromosomes from cytoplasmic proteins following nuclear envelope breakdown (Yasuda and Maul, 1990), (3) protecting chromosomes from sticking to one another (Cuylen et al., 2016) and (4) acting as a scaffold to distribute nucleolar proteins required for post-mitotic nucleolar reactivation (Montgomery, 1900; Metz, 1934). Even though experimental evidence is available to support each of these hypotheses, the functional significance of the chromosome periphery during mitosis remains elusive.

Most chromosome periphery proteins are associated with the nucleolus or the nucleoplasm during interphase until the transition from G2 to mitosis, when, upon nuclear envelope breakdown, they accumulate at the chromosome periphery in a sequential manner. Among the chromosome periphery components, there are many ribonucleoproteins, RNAs (Gautier et al., 1992; Hernandez-Verdun and Gautier, 1994; Van Hooser et al., 2005) and nucleolar proteins, including fibrillarin, nucleolin (NCL), B23 (nucleophosmin or NPM1), peripherin and Ki-67 (MKI67) (Booth and Earnshaw, 2017; Hernandez-Verdun and Gautier, 1994; Jansen et al., 1991; Tollervey and Kiss, 1997; Yasuda and Maul, 1990). Despite the fact that the chromosome periphery originally received little attention other than in studies of nucleolar inheritance (Gautier et al., 1992; Hernandez-Verdun, 2006; Hernandez-Verdun and Gautier, 1994; Hernandez-Verdun et al., 2013; Muro et al., 2010), in the past several years, interest in this structure has substantially increased.

Ki-67 is one of the earliest proteins associated with the chromosome periphery from early prometaphase to telophase (Van Hooser et al., 2005) and it is essential for the formation of the chromosome periphery (Booth et al., 2014). In fact, Ki-67 depletion has been found to lead to the disappearance of the chromosome periphery, with significant effects on the distribution of nucleolar components during mitosis (Booth et al., 2014). Interestingly, mitotic chromosomes lacking the perichromosomal layer tend to aggregate, suggesting the interesting hypothesis that Ki-67 forms a steric and electrostatic coating on chromosomes by acting as a biological surfactant that is required to maintain the individuality of each mitotic chromosome (Cuylen et al., 2016). Although Ki-67 appears to be a master organiser of this compartment, many other proteins are present in the layer. Whether they fulfil specific functions in chromosome structure or dynamics is not generally known, although the recent discovery of the NOL11–WDR43–cirhin (NWC) complex and demonstration that it is required for Aurora B recruitment to chromosomes (Fujimura et al., 2020) raises the possibility that other components might also have important roles in chromosome segregation.

Several studies have analysed the effects of nucleolin and B23 depletion in human cells. These led to diverse phenotypes, including defects in chromosome alignment and segregation with micronucleus formation, mitotic delay, increased apoptosis and abnormal nucleolar architecture (Ugrinova et al., 2007; Amin et al., 2007; Ma et al., 2007). In contrast, fibrillarin depletion did not induce the same phenotype but appeared to be involved in maintaining nuclear shape integrity (Amin et al., 2007).

Late in mitosis, the nucleolar proteins associated with the chromosome periphery become diffuse in the cytoplasm and associate in particles called nucleolus-derived foci (NDFs) (Hernandez-Verdun, 2011; Hernandez-Verdun et al., 2013; Muro et al., 2010; Dundr et al., 2000). Afterwards, components of the NDFs fuse to form pre-nucleolar bodies, which are distributed throughout early G1 nuclei (Dimario, 2004; Dundr et al., 2000; Olson and Dundr, 2005). These dynamics are important for the reformation of a functional nucleolus.

The diverse mitotic phenotypes associated with perturbations of the perichromosomal layer raise the possibility that sub-compartments of this structure might have specific mitotic functions. It is therefore important to identify and dissect various subcomplexes to understand how these chromosome structures are coordinated.

In this study, we identified coiled-coil domain-containing protein 86 (CCDC86, also known as cyclon) as a new component of the perichromosomal layer. We show that its depletion causes defects in chromosome alignment and segregation without perturbing the localisation of Ki-67 at the chromosome periphery in early mitosis, and alters the composition of NDFs in anaphase and telophase. This finding, taken together with the discovery of the NWC complex (Fujimura et al., 2020), reveals that indeed some subcomplexes of this layer might have specific mitotic functions. We also discovered that *CCDC86* is a MYCN-regulated gene, the expression levels of which are of prognostic value in Neuroblastoma patients.

## RESULTS

### CCDC86 is a novel perichromosomal layer component

**Fig. 1.**
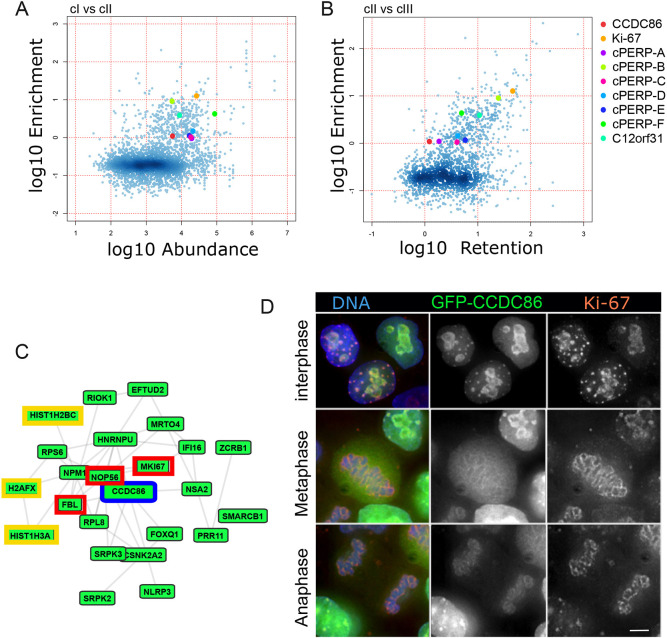
**CCDC86 is a nucleolar protein associated with the chromosome periphery.** (A,B) Two-dimensional scatter plot showing the fold-change (log10) of protein abundance on mitotic chromosomes (cI, A) or *in vitro* exchange on chromosomes (cIII, B) versus enrichment on chromosomes (cII) generated in previous proteomics analysis (Ohta et al., 2010). The colours indicate the different chromosome periphery proteins as indicated in the legend. The colour intensities, ruled by the smooth density plot in R, indicate the population densities. (C) CCDC86 interactome (GPS-Prot – human protein–protein interactions). Known nucleolar proteins are highlighted in red and histones in yellow. Interacting proteins are coloured green. (D) A GFP–CCDC86 construct (green) was transfected in HeLa cells. Cells were fixed and stained for Ki-67 (red) and DNA (blue). Images representative of >10 independent experiments showing GFP–CCDC86 localisation in interphase (top row), metaphase (middle row) and telophase (bottom row). Scale bar: 5 µm.

In order to understand the composition and the function of the perichromosomal layer in mitosis, we need to be able to identify each subcomplex and assess its specific contribution. We have previously shown that depletion of Ki-67 causes a dramatic phenotype as it is at the top of the hierarchy and is required for the assembly of the entire chromosome periphery (Booth et al., 2014). To identify novel proteins that could be part of this structure and classify them into different subcomplexes, we reanalysed the mitotic chromosome proteome (Ohta et al., 2010, 2011). As shown before, bona fide mitotic chromosome proteins cluster when the proteome is analysed using the ‘Abundance’ on chromosomes classifier versus the ‘Enrichment’ classifier (Ohta et al., 2010). Ribosomal and nucleolar proteins have a lower retention coefficient, possibly indicating significant binding to chromosomes from cytosol during the incubation as shown for Ki-67, cPERP-A–F and c12orf31, all chromosome periphery proteins (Fig. 1A,B) (Ohta et al., 2011). This could reflect the nature of these complexes, which undergo major dynamic re-localisation transitions during mitosis.

Based on these two classifiers, we identified as a novel candidate chromosomal protein, CCDC86 (Fig. 1A,B, red circles). The Gene Ontology for this protein includes three cellular components: ‘nucleus’ (GO:0005634), ‘nucleoplasm’ (GO:0005654) and ‘nucleolus’ (GO:0005730). Using the ‘GPS-Prot: Data Visualization for Protein–Protein Interactions’ tool (http://gpsprot.org/; Fahey et al., 2011), we analysed the known interactome for CCDC86. GPS-Prot integrates human protein interactions derived from publicly available databases including MINT (https://mint.bio.uniroma2.it/) and BioGRID (https://thebiogrid.org/). CCDC86 shows interactions with known nucleolar proteins that belong to the mitotic perichromosomal layer, including Ki-67, fibrillarin, NOP56 and several histones (Fig. 1C). We therefore generated GFP fusion constructs (both N- and C-terminally tagged) of CCDC86 for localisation studies.

In HeLa cells, both fusion proteins localised to the nucleolus in interphase. They were mainly dispersed in the cytoplasm in mitosis but enriched at the periphery of the chromosomes. They were highly enriched at the chromosome periphery during anaphase (Fig. 1D). Interestingly, co-staining with anti-Ki-67 antibodies revealed that Ki-67 and CCDC86 always colocalised in interphase and during mitotic exit, but only partially during prometaphase and metaphase (Fig. 1D, middle panel). Therefore, this novel protein could be an interesting candidate to further dissect the individual functions of the chromosomal periphery subcomplexes.

### CCDC86 enrichment to the chromosomes depends on Ki-67 and its first AT-hook like domain

**Fig. 2.**
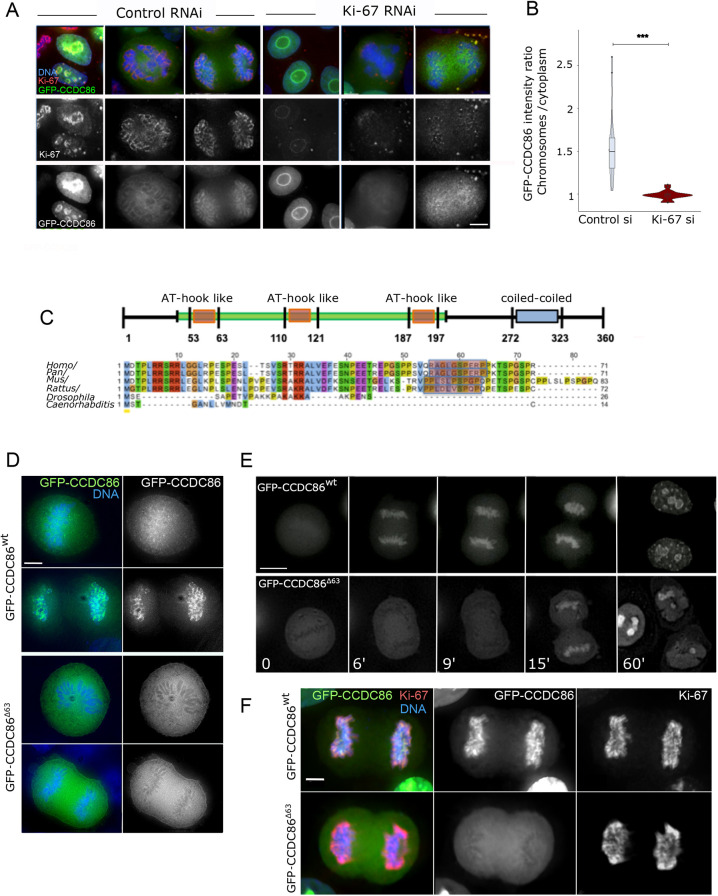
**CCDC86 requires Ki-67 and the first AT-hook domain for its recruitment to mitotic chromosomes.** (A) HeLa cells were transfected with a GFP–CCDC86 construct (green) and treated with control or Ki-67 siRNA oligonucleotides. Cells were then fixed and stained for Ki-67 (red) and DNA (blue). Representative images of interphase (left panels), prometaphase (middle panels) and anaphase (right panels) cells are shown for both control and Ki-67 RNAi. Scale bar: 10 µm. (B) GFP–CCDC86 enrichment on anaphase chromosomes. The values represent the ratio of the GFP intensity values on chromosomes to those in the cytoplasm. *n*=19 anaphase cells for control siRNA (si) and *n*=20 anaphase cells for Ki-67 siRNA from two independent experiments. The data were statistically analysed with a Wilcoxon signed rank test. ***P<0.0001. Boxes show the 25–75th percentiles, the whiskers show the upper and lower adjacent values and the median is marked with a line. (C) Scheme of the CCDC86 protein (top) showing the position of the three AT-hook-like domains and the coiled-coiled region. Alignment of the N-terminal regions of the protein from various species (bottom). Shadowed in red are the sequences of the first AT-hook. (D) HeLa cells were transfected with a GFP–CCDC86^wt^ construct or the mutant lacking the first 63 amino acids (GFP–CCDC86^Δ63^) (green), then fixed 24 h post transfection. Representative metaphase and anaphase cells are shown. Scale bar: 5 µm. (E) Live-cell imaging of HeLa cells transfected with the GFP–CCDC86^wt^ or GFP–CCDC86^Δ63^ constructs. Scale bar 20 µm. (F) HeLa cells were transfected with either the GFP–CCDC86^wt^ GFP–CCDC86^Δ63^ constructs (green), then fixed 24 h post transfection and stained for Ki-67 (red). Representative anaphase cells are shown. Scale bar 5 µm. Images are representative of >10 independent experiments.

As most perichromosomal layer proteins depend on Ki-67 for their localisation (Booth et al., 2014; Stenstrom et al., 2020), we tested whether Ki-67 RNA interference (RNAi) would also impair the localisation of CCDC86. As predicted, Ki-67 RNAi strongly diminished the enrichment of CCDC86 at the periphery of the chromosomes both in early mitosis and during mitotic exit (Fig. 2A,B). Therefore, CCDC86 recruitment to the chromosomes is mediated or stabilised by Ki-67. However, interactome analysis (Fig. 1C) showed that histones are also part of the CCDC86 interaction network. Moreover, domain analyses of CCDC86 revealed the presence of three conserved AT-hook-like motifs in the protein (Fig. 2C). These motifs are found in a variety of DNA-binding and DNA-remodelling proteins, including the high-mobility group (HMG) proteins and the BRG1 protein (part of the SWI/SNF remodelling complex) that have a preference for AT-rich regions (Reeves and Nissen, 1990). We therefore tested whether these motifs were involved in the localisation of CCDC86.

We generated GFP-tagged truncated proteins by deleting either the first (GFP–CCDC86^Δ63^), the first and second (GFP–CCDC86^Δ121^), or all three AT-hook-like domains of CCDC86 (GFP–CCDC86^Δ197^). All these mutant fusion proteins failed to localise to the chromosomes when analysed as transient transfections in HeLa cells (Fig. 2D; Fig. S1A). The localisation of the mutant in which all three AT-hook motifs were deleted showed the same localisation patterns as GFP–CCDC86^Δ63^ (Fig. S1A). To gain more information about the dynamic behaviour of these proteins, we conducted live-cell imaging using either a GFP-tagged CCDC86 wild-type protein (GFP–CCDC86^wt^) or a mutant protein lacking the first AT-hook (GFP–CCDC86^Δ63^). Although GFP–CCDC86^wt^ was first seen on the chromosomes in metaphase cells and then strongly accumulated on anaphase chromosomes (Fig. 2E, top panels), the mutant counterpart failed to localise on the chromosomes in metaphase and anaphase (Fig. 2E, bottom panels). GFP–CCDC86^Δ63^ accumulated on chromosomes only several minutes after anaphase onset. This is the time when the nuclear envelope is reforming and nuclear import starts. Later in G1 (Fig. 2E, 60 min), the mutant protein accumulated in the nucleoli. Moreover, expression of GFP–CCDC86^Δ63^ did not displace Ki-67 from its normal localisation at the chromosome periphery (Fig. 2F). These data altogether suggest that CCDC86 recruitment to the chromosome periphery is downstream of Ki-67 and requires the first AT-hook-like domain of CCDC86.

### CCDC86 depletion causes the formation of abnormal NDFs

**Fig. 3.**
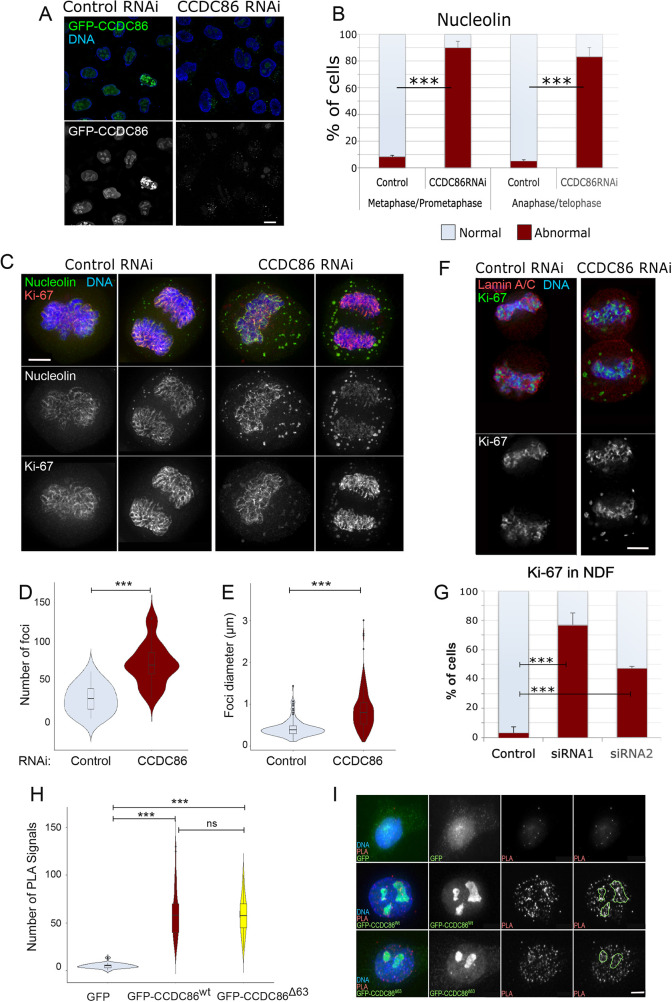
**Depletion of CCDC86 compromises the mitotic localisation of nucleolin and Ki-67.** (A) HeLa cells were transfected with a GFP–CCDC86 construct (green) and treated with control or CDCD86 RNAi oligo (#1) for 48 h. The cells were fixed and counterstained with DAPI (blue). Representative images showing cells with GFP–CCDC86 (green) in control RNAi and no GFP signal in CCDC86 RNAi. Scale bar: 5 µm. (B) Quantification of cells displaying an abnormal nucleolin pattern in metaphase/prometaphase or anaphase/telophase cells from the experiment in C. The graph shows the average of three independent experiments and the error bars represent the s.d. Fifty cells from each replica and condition were analysed. Statistical analyses were conducted using the χ^2^ test. (C) HeLa cells were treated with either control or CCDC86 siRNA for 48 h, then fixed and stained for nucleolin (green), Ki-67 (red) and DNA (blue). Representative images of a metaphase cell (left) and an anaphase cell (right) after each RNAi treatment from three independent experiments are shown. Scale bar: 5 µm. (D) Violin plots representing the number of nucleolin foci in anaphase/telophase cells from the experiment in C that were treated with control or CCDC86 siRNAs. Statistical analyses were conducted using a Wilcoxon signed-rank test. (E) Violin plots representing the diameter of nucleolin foci in anaphase/telophase cells from the experiment in C that were treated with control or CCDC86 siRNA. Statistical analyses were conducted using a Wilcoxon signed-rank test. (F) HeLa cells were treated with either control or CCDC86 siRNA (oligo #1 or oligo #2) for 48 h and then fixed and stained for Ki-67 (green), lamin A/C (red) and DNA (blue). Representative images of telophase cells (as judged by the recruitment of lamin A/C around the chromosomes) after each RNAi treatment are shown. Scale bar: 5 μm. (G) Quantification of cells displaying an abnormal Ki-67 pattern in telophase cells from the experiment in F. The graph shows the average or three experiments and the error bars represent the s.d. Statistical analyses were conducted using the χ^2^ test. (H) HeLa cells were transfected with either GFP, GFP–CCDC86^wt^ or GFP–CCDC^Δ63^ constructs. At 24 h post transfection, cells were fixed and subjected to PLA using antibodies against GFP and Ki-67. The graph represents the number of PLA signals per cell in the three conditions. Statistical analyses were conducted using a Wilcoxon signed rank test. (I) Representative images of the experiment in H. The green masks in the fourth column represent the localisation of the nucleoli. Scale bar: 5 µm. For the violin plots, the boxes indicate the 25–75th percentiles, the whiskers show the upper and lower adjacent values and the median is marked with a line. ns, not significant; ***P<0.0001.

**Fig. 4.**
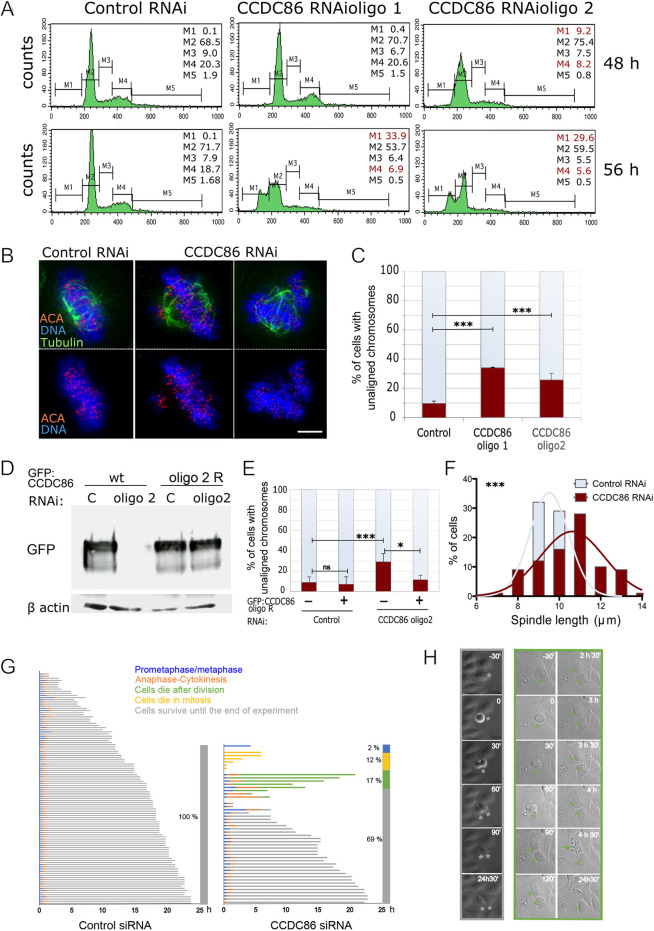
**Depletion of CCDC86 compromises normal mitotic progression.** (A) HeLa cells were transfected with control or CCDC86 siRNAs for 48 or 56 h. The cells were then harvested and subjected to cell cycle analyses by flow cytometry. The percentages of cells in each gated interval are shown: M1 (sub-G1), M2 (G1), M3 (S), M4 (G2) and M5 (>2N). Highlighted in red are the stages in which there is a significant deviation from the control distribution. (B) HeLa cells were treated with either control or CCDC86 siRNAs (oligo #1 or oligo #2) for 48 h and then fixed and stained for tubulin (green), anti-centromere antibody (ACA) (red) and DNA (blue). Representative images of normal metaphase cells with aligned chromosomes (left) and cells with unaligned chromosomes (right) after each RNAi treatment. Scale bar: 5 μm. (C) Quantification of mitotic cells with unaligned chromosomes from the experiment in B. The graph shows the average of three experiments and the error bars represent the s.d. *n*=166 (control), 228 (oligo 1) and 182 (oligo 2). Statistical analyses were conducted using the χ^2^ test. ***P<0.0001. (D) HeLa cells were transfected with a GFP–CCDC86^wt^ or the siRNA-resistant construct (oligo 2 R) together with control or CCDC86 (oligo2) siRNAs for 48 h. Cells were collected and whole-cell lysates were subjected to SDS-PAGE and immunoblotting using an anti-GFP or anti-β-actin antibody. Images are representative of >10 independent experiments. (E) HeLa cells were treated as in D, then fixed and immunostained as in B. The graph shows the quantification of mitotic cells with unaligned chromosomes in the different conditions. The graph shows the average of three experiments and the error bars represent the s.d. *n*=166 (control untransfected), 130 (control transfected), 510 (CCDC86 RNAi untransfected), 170 (CCDC86 RNAi transfected). Statistical analyses were conducted using the χ^2^ test. ns, not significant; *P<0.05; ***P<0.0001. (F) Distribution of the spindle lengths obtained from the experiment in B. Pole-to-pole distances were obtained from bipolar metaphase cells when the spindle poles were in the same focal plane. *n*=35. Statistical analyses were conducted to compare the spindle lengths between control and CCDC86 RNAi cells by Mann–Whitney test. (G) Cell fate profiles, as determined by time-lapse microscopy, of cells treated with control or CCDC86 siRNAs. *T*_0_ represents the time each cell rounded up and images were acquired every 30 min. The different fates are colour coded as indicated in the legend. The bar at the right of each graph indicates the percentage of cells that followed each fate. Each horizontal line represents a single cell. *n*=108 for control siRNA, *n*=47 for CCDC86 siRNA. (H) Frames from time-lapse imaging of CCDC86 siRNA cells that divided and survived until the end of the experiment (left, grey frames and grey asterisks) and cells that died after division (right, green frames and green asterisks; the green arrow indicates the daughter cell that died).

We next analysed the mitotic phenotypes of cells in which CCDC86 was depleted. We used two siRNAs that efficiently depleted the protein by 48 h of treatment (Fig. 3A, Fig. 4B) and first analysed the localisation of both Ki-67 and nucleolin. Interestingly, the two proteins were present at the mitotic chromosome periphery both in early mitosis and during mitotic exit, but they were also observed in cytoplasmic foci that resemble NDFs in both stages of mitosis. Although nucleolin is found in NDFs in normal cells (Dundr et al., 2000), the foci were much bigger (Fig. 3C,E) and more abundant (Fig. 3C,D) after CCDC86 depletion, and were also present in prometaphase and metaphase (Fig. 3B–E), which is not normal. Moreover, a larger number of foci formed in CCDC86-depleted cells and, interestingly, these were excluded from the spindle region (Fig. S1B). Remarkably, after CCDC86 depletion, Ki-67 was also detected in NDFs. This has never been observed in unperturbed cells. We therefore quantified the frequency of telophase cells in which Ki-67 was present in the abnormal NDFs using lamin A/C co-staining in order to score cells at the same stage of mitotic exit. The results, using two independent siRNAs, revealed a significant increase in the number of telophase cells with NDFs containing Ki-67 after CCDC86 depletion (Fig. 3F,G).

CCDC86 has been identified as a Ki-67 interactor upon Ki-67 pulldown followed by mass spectrometry (Sobecki et al., 2016). Therefore, we wanted to investigate whether the reported interactions between Ki-67 and CCDC86 could also be confirmed in cells and whether the interaction was confined to a specific subcellular compartment. To look for association between Ki-67 and CCDC86 *in vivo*, we used a proximity ligation assay (PLA) approach. We used asynchronous HeLa cells transfected with either GFP, GFP–CCDC86^wt^ or GFP–CCDC86^Δ63^ and conducted PLA experiments using anti-GFP and anti-Ki-67 antibodies. The analyses showed numerous PLA signals between Ki-67 and both versions of GFP–CCDC86 but not with Ki-67 and GFP (Fig. 3H,I). These signals were distributed throughout the nuclear space, but some enrichment could be detected at the periphery of the nucleoli (Fig. 3I).

We therefore conclude that CCDC86 likely interacts with Ki-67 *in vivo* within the nucleus, with some enrichment at the nucleolar periphery, and that the first AT-hook domain of CCDC86 is apparently not essential for this interaction. However, because we did not conduct the PLA experiments in a CCDC86 knockdown background, we cannot exclude that a dimer or a higher-order complex including also the endogenous wild-type protein could yield a positive PLA result.

### CCDC86 is important for chromosome segregation

Having identified CCDC86 as a novel component of the chromosome periphery, the depletion of which does not abolish the localisation of known perichromosomal layer proteins such as nucleolin and Ki-67, we wanted to check whether depletion of this protein was associated with chromosome defects in mitosis. We first analysed the cell cycle profile of cells depleted of CCDC86 by flow cytometry using the two different siRNA oligonucleotides (oligos 1 and 2) at 48 and 56 h of treatment. The analyses showed an increase in the sub-G1 population (indicative of apoptosis) and a decrease in the G1 and G2 populations following transfection with either oligonucleotide (Fig. 4A). This could suggest that cells lacking CCDC86 enter apoptosis during or after cell division.

We therefore analysed the chromosomal phenotype of control and CCDC86-depleted mitotic cells. This analysis revealed a significant increase in cells in which most of the chromosomes were aligned at the metaphase plate apart from a few misaligned chromosomes (Fig. 4B,C). Importantly, this phenotype was rescued by expressing a CCDC86 cDNA resistant to oligo 2 (Fig. 4D,E). Chromosome attachment errors are also often associated with spindle-length variations (Nannas et al., 2014); we therefore measured the spindle length in metaphase cells with a bipolar spindle. Indeed, CCDC86 RNAi cells had significantly longer spindles than cells treated with control RNAi (Fig. 4F).

Based on the flow cytometry data and mitotic phenotypes, we then wanted to check whether mitotic progression was altered in cells depleted of CCDC86 and the fate of cells after division. We therefore conducted live-cell imaging of cells treated with control or CCDC86 siRNAs and imaged cells for 25 h every 30 min. For all the cells that entered mitosis, we recorded the time taken by each cell from rounding up to cell elongation (anaphase and/or telophase), the duration between anaphase onset and the completion of division, and the outcome of the daughter cells (if alive until the end of the experiment or the time point at which they died after division).

The analysis of these experiments clearly showed that cells treated with control RNAi had an early mitosis length of 44±18 min (indicated as mean±s.d.) and a mitotic exit duration (anaphase to cytokinesis) of 30 min, and that all cells survived after division until the end of the experiment. On the contrary, cells treated with CCDC86 siRNA had an early mitosis length of 90±282 min and a mitotic exit duration of 67±60 min, and, of the cells that entered mitosis, 12% died in mitosis and 17% died after division (either both daughter cells or one of them) (Fig. 4F,G). However, we noticed that some cells also died without dividing during the imaging time (compare the beginning and the end of the experiments in Fig. S2). At this point, we do not know whether those cells died because they divided before we started imaging or whether CCDC68, in addition to being required for a successful mitosis, also has some essential functions in interphase. Interestingly, the percentage of cells that died during or after mitosis (31%) is very similar to the percentage of cells showing mitotic defects (Fig. 3C). Taken together, these data show that CCDC86 is required for the execution of an error-free mitosis.

### CCDC86 is a MYCN-regulated gene with a prognostic value for neuroblastoma patients

Although very little is known about CCDC86 biology, this protein has already emerged as an important predictor of cancer resistance and outcome in several studies (Bouroumeau et al., 2021; Emadali et al., 2013). Furthermore, CCDC86 was identified to be an autonomous tumour growth driver that cooperates with MYC to drive aggressive lymphoma growth *in vivo* (Emadali et al., 2013).

**Fig. 5.**
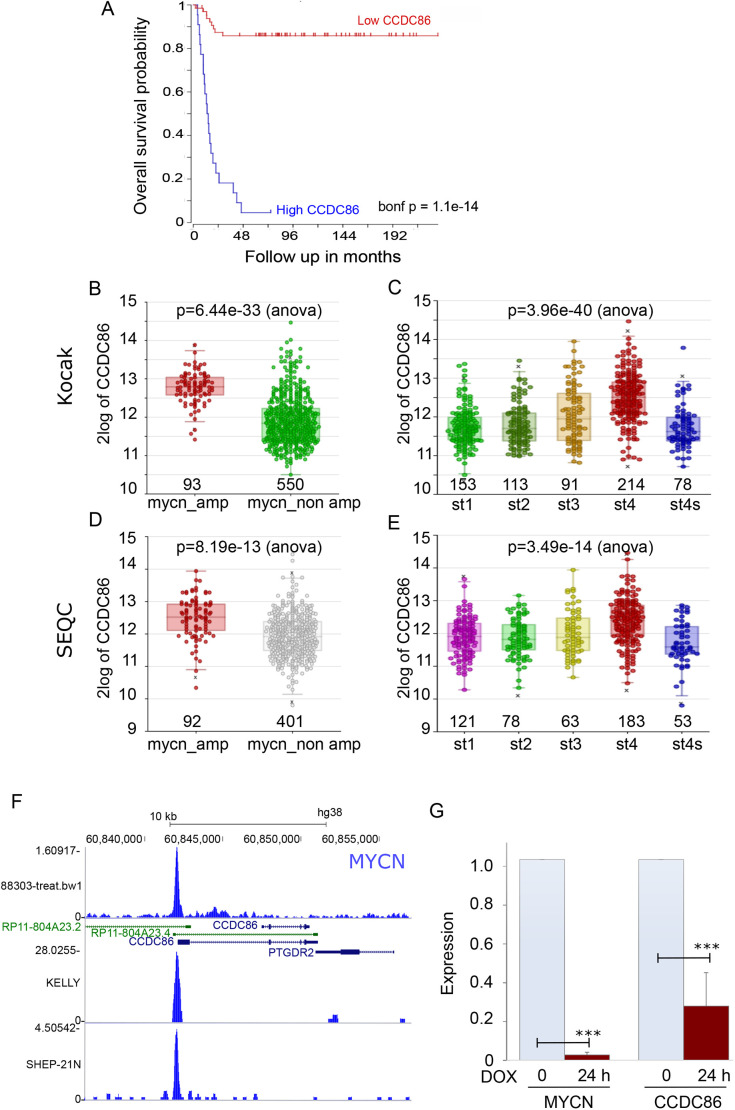
**CCDC86 expression is regulated by MYCN and represents a prognostic marker for neuroblastoma patients.** (A) Kaplan–Meier curve (survival probability in months) of neuroblastoma patients expressing low (red curve) or high (blue curve) levels of CCDC86. (B) CCDC86 expression levels in neuroblastoma patients with (red) and without (green) *MYCN* amplification (Kocak cohort). (C) CCDC86 expression levels in neuroblastoma patients with different stages (st) of cancer (Kocak cohort). (D) CCDC86 expression levels in neuroblastoma patients with (red) and without (grey) *MYCN* amplification (SEQC cohort). (E) CCDC86 expression levels in neuroblastoma patients with different stages of cancer (SEQC cohort). In B–E, the numbers indicate the sample sizes. (F) MYCN chromatin immunoprecipitation sequencing profiles at the *CCDC86* locus in different neuroblastoma cell lines. (G) qPCR analyses of *MYCN* and *CCDC86* expression in TET21-N cells without (0) (light blue bars) and with 24 h doxycycline (DOX) treatment (red bars). The data represent the average of three independent experiments. Error bars indicate the s.d. Datasets were analysed by a two-tailed unpaired Student's *t*-test. ***P<0.0001.

Neuroblastoma is a tumour in which amplification of the *MYCN* gene correlates with poor prognosis (Huang and Weiss, 2013). We therefore analysed survival data for neuroblastoma patients in the Kocak and SEQC cohorts (GSE45547 and GSE49710, respectively) using the R2 genomics platform (http://r2.amc.nl) and related to *CCDC86* expression. As the Kaplan–Meier curves show, high levels of *CCDC86* strongly correlate with a very low survival rate in this type of cancer (Fig. 5A). We then stratified the patients in each cohort based on whether or not *MYCN* is amplified in the tumours and based on their stages. The results obtained from both cohorts are very similar and clearly show that high expression of *CCDC86* is found in patients with *MYCN* amplifications (Fig. 5B,D) and the expression level of *CCDC86* positively correlates with tumour stage progression and malignancy (Fig. 5C,E). In fact, in stage 4 tumours (which are not metastatic), *CCDC86* presents low expression. These data suggest that *CCDC86* expression could be driven by MYCN and, even in neuroblastoma patients, represents a very useful prognostic marker.

We therefore analysed MYCN occupancy at *CCDC86* promoters in different MYCN-amplified cell lines using the UCSC genome browser. In all profiles analysed, a clear peak for MYCN was present at the *CCDC86* promoter (Fig. 5F). To confirm the dependency of *CCDC86* expression on MYCN, we then used the neuroblastoma cell line TET21-N, in which *MYCN* expression can be modulated by doxycycline (Lutz et al., 1996). Addition of doxycycline to the culture decreases the expression of *MYCN* after 24 h (Fig. 5G, left) as evaluated by quantitative PCR (qPCR). Cell viability is not affected by MYC expression in this system. We then tested the expression of CCDC86 in the same conditions; strikingly, repression of *MYCN* significantly decreases CCDC86 expression. These data therefore confirm that *CCDC86* expression is regulated by MYCN in neuroblastoma, and that CCDC86 is a useful prognostic marker in this type of cancer.

## DISCUSSION

The chromosome periphery is an outer layer coating the surface of mitotic chromosomes and contains a large number of proteins with diverse functions during interphase. The list of components for this compartment is still growing, as more proteins associated with the chromosome periphery are been identified by proteomics screens of isolated chromosomes (Ohta et al., 2011; Takata et al., 2007; Stenstrom et al., 2020). Despite the ever-growing list of components, the chromosome periphery is not very well characterised with regard to either its composition or its function. It is also unclear whether the chromosome periphery is functionally a single domain or whether it represents an assembly of multiple partially overlapping subcomplexes that collaborate to execute various functions of this structure. The diversity of chromosome periphery-associated proteins (Van Hooser et al., 2005) reveals a need for better understanding of the composition and function of this complex compartment.

We have analysed the mitotic chromosome proteome with the goal of identifying novel components of the chromosome periphery (Ohta et al., 2011). This approach led us to study a novel chromosomal protein, CCDC86. CCDC86 was previously described as a downstream effector of IL-3 signalling upon cytokine induction in haematopoietic stem cells (Hoshino and Fujii, 2007). CCDC86 was also shown to play a role in T-cell activation-induced cell death (Saint Fleur et al., 2009) and studies have also linked CCDC86 expression with cancer progression (Emadali et al., 2013; Bouroumeau et al., 2021). However, CCDC86 biology still remains largely unexplored.

We discovered that CCDC86 exhibited a localisation pattern that, in many ways, resembles that of Ki-67 and other chromosomal periphery proteins (Amin et al., 2007; Ma et al., 2007). A GFP fusion protein of CCDC86 localised to the nucleolus during interphase and was recruited to the chromosome periphery during mitosis. However, CCDC86 exhibited its most prominent association with the chromosome periphery late in mitosis. The weaker association of CCDC86 with mitotic chromosomes in early mitosis could be the result of a dynamic behaviour of the protein when phosphorylated. In fact, CCDC86 presents several phosphorylation sites that are affected by nocodazole treatment (as shown in the PhosphoSite database; https://www.phosphosite.org/).

Ki-67, a major determinant of the assembly of chromosome periphery (Booth et al., 2014), is required for the recruitment of CCDC86 to the chromosomes. RNAi-mediated depletion of Ki-67 prevented CCDC86 association with the chromosome periphery. Interestingly, a mutant version of GFP–CCDC86^Δ63^ lacking the first AT-hook domain also failed to localise to mitotic chromosomes, even though this mutant protein could still interact with Ki-67 *in vivo* (at least as assessed by PLA). This observation could indicate that CCDC86 has a chromosome periphery-targeting module that does not solely depend on its interaction with Ki-67. In interphase, upon Ki-67 RNAi, GFP–CCDC86 is diffuse in the nuclear space with enrichment at the periphery of the nucleoli, suggesting that its nuclear foci accumulation is also Ki-67 dependent. Combining these results with the Gene Ontology data and the CCDC86 protein–protein interactome from GPS-Prot, we conclude that CCDC86 is a bona fide chromosome periphery-associated protein.

**Fig. 6.**
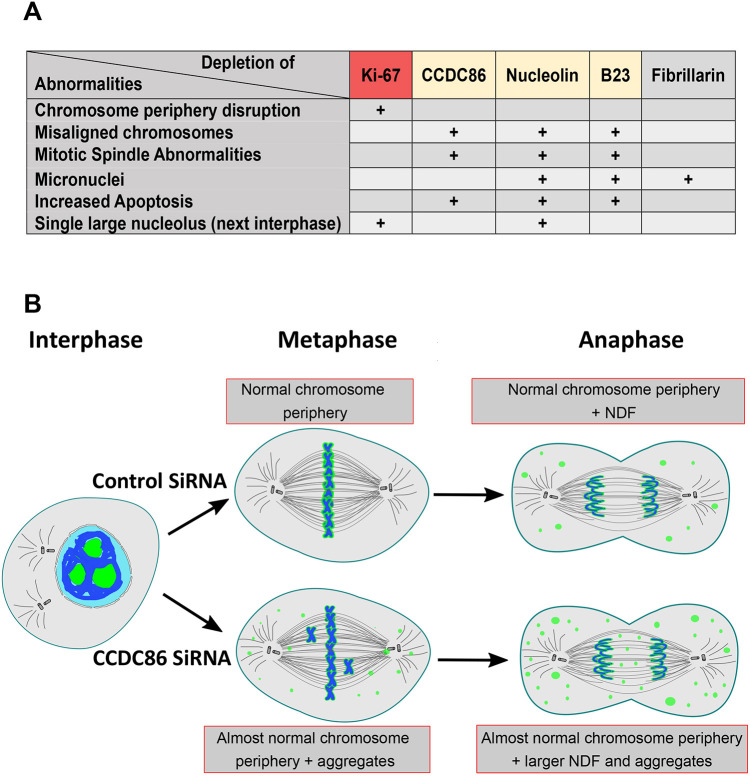
**Model for mitotic functions of subcomplexes of the chromosome periphery.** (A) Table showing the major phenotypes associated with the depletion of the indicated proteins (Amin et al., 2007; Booth et al., 2014; Ma et al., 2007; Ugrinova et al., 2007). (B) Graphical summary of CCDC86 function in mitosis.

CCDC86 depletion led to a novel phenotype in which Ki-67 and nucleolin could still accumulate at the chromosome periphery but also formed abnormal cytoplasmic NDF-like foci. This is the first time that Ki-67 has been observed in abnormal NDFs. For nucleolin, these foci were more abundant and increased in number compared to control cells, and they were atypically present in prometaphase (Ochs et al., 1983). Taken together, these observations reveal that upon depletion of CCDC86, the chromosome periphery compartment is maintained, but some of its components exhibit additional abnormal interactions (Fig. 6B).

Ki-67 association with the chromosome periphery is more dynamic in early mitosis and more stable from mitotic exit onwards (Endl and Gerdes, 2000; MacCallum and Hall, 2000; Schlüter et al., 1993; Takagi et al., 2014). During mitosis, Ki-67 is hyperphosphorylated in a CDK1-dependent manner. These phosphorylated sites decrease the affinity of Ki-67 for the DNA until anaphase onset, at which point protein phosphatase 1 (PP1) reverses the phosphorylation on these sites, thus increasing Ki-67 association with the chromatin (Saiwaki et al., 2005; Takagi et al., 2014), and might also play a role in regulating Ki-67 surfactant properties (Cuylen et al., 2016; Cuylen-Haering et al., 2020; Stamatiou and Vagnarelli, 2021). Staurosporine treatment of metaphase-arrested cells causes the disassociation of Ki-67 and B23 from the chromosome periphery and the formation of abnormal cytoplasmic foci (NDF-like foci) (MacCallum and Hall, 2000). Interestingly, CCDC86 depletion led to a similar phenotype. We hypothesise that Ki-67 dynamics might not only be regulated by its phosphorylation status, but also by specific protein–protein interactions.

We observed errors in chromosome alignment in cells depleted of CCDC86. This phenotype was specific as it was rescued by transient expression of a siRNA-resistant CCDC86 construct. In addition, cells lacking CCDC86 displayed a significantly longer spindle length compared to that of control cells and an increase in the frequency of apoptosis, possibly as a consequence of mitotic defects. Depletion of CCDC86 caused an increase in the number of cells that had a prolonged mitosis, with a high percentage of cells dying in mitosis or after division. These observations, combined with the partial disassociation of nucleolin from chromosomes after CCDC86 depletion, suggest that chromosome misalignment and mitotic spindle defects observed in these cells might be consequences of the disassociation of nucleolin and, to a certain extent, of B23 from chromosomes. In fact, depletion of nucleolin or B23 leads to chromosome misalignment, defects in mitotic spindle formation and apoptosis (Amin et al., 2007; Ma et al., 2007; Ugrinova et al., 2007). Nucleolin appears to be upstream of B23, as B23 disappears from the chromosome periphery after nucleolin depletion (Ma et al., 2007). Our data are consistent with CCDC86 being upstream of B23 for the regulation of mitotic spindle formation and correct microtubule attachment.

Interestingly, these phenotypes are masked when Ki-67 is depleted, and the entire chromosome periphery layer is removed (Booth et al., 2014; Cuylen et al., 2016). Being able to remove only a subset of proteins can thus reveal some different functions that are embedded in this compartment (Fig. 6A). The classifications of the phenotypes appear to suggest the existence of a cluster of functions for the chromosome periphery subcomplexes. In fact, the recently identified NWC complex, which is part of the mitotic chromosome periphery, is required for the centromeric enrichment of Aurora B and the downstream phosphorylation of histone H3 at threonine 3 (Fujimura et al., 2020).

We have also shown that the expression of the *CCDC86* gene is regulated by MYCN and has prognostic value in neuroblastoma. In this respect, considering that CCDC86 depletion induces apoptosis, it would be interesting to evaluate in the future whether this protein could also represent a clinically relevant drug target. In conclusion, our study represents an additional step towards understanding the complexity and functional significance of the chromosome periphery in mitosis and cell cycle progression.

## MATERIALS AND METHODS

### Cell culture, cloning and transfection

HeLa Kyoto cells were grown in Dulbecco's modified Eagle medium (DMEM; Gibco) supplemented with 10% foetal bovine serum (FBS; Labtech) and 1% penicillin-streptomycin (Invitrogen, Gibco) at 37°C with 5% CO_2_. CCDC86 was cloned in pEGFP-N1 (Takara) and pEGFP-C1 (Takara) by PCR from HeLa cell cDNA using the following primers: Fw, 5′-CGGATCCGGCGGGATGGATACACCGTTAAGG-3′, and Rev, 5′-CAGAATTCGGATCTTGGCTGC-3′. The oligonucleotide-resistant mutation was created as follows: 5′-AGATTCTCCCAGATGTTACAAGAC-3′.

For the siRNA treatments, HeLa cells in exponential growth were seeded in six-well plates, transfected using Polyplus JetPrime (PEQLAB, Southampton, UK) with the appropriate siRNA oligonucleotides (50 nM) and analysed at the times indicated in the experiments. The siRNAs were obtained from Sigma-Aldrich: control: 5′-CGUACGCGGAAUACUUCGA-3′; CCDC86 Oligo 1, SASI_Hs01_00120887; and CCDC86 Oligo 2, SASI_Hs01_00120888. For the rescue experiments, 400 ng of the wild-type or oligonucleotide-resistant constructs were used.

The TET21-N cell line (Lutz et al., 1996) was kindly gifted by Prof. Arturo Sala (Brunel University London, UK). Neuroblastoma cells were cultured in DMEM supplemented with 10% FBS and 100 U/ml penicillin/streptomycin. TET21-N cells were routinely maintained in medium containing 0.2 mg/ml G-418 (11811031, Gibco) and 0.15 mg/ml hygromycin B (10453982, Invitrogen). To switch off *MYCN* expression, cells were cultured in the presence of 1 µg/ml doxycycline (D9891, Sigma-Aldrich).

### Immunoblotting

Whole-cell lysates were loaded onto polyacrylamide gels. SDS-PAGE and immunoblotting were performed following standard procedures. The following primary antibodies were used: anti-GFP (1:2000, ChromoTek, PABG1-2) and anti-β-actin (1:1000, Invitrogen, clone 15G5A11/E2, MA1-140). The following secondary antibodies were used: horseradish peroxidase-conjugated anti-mouse IgG (1:5000, Thermo Fisher Scientific, 31444).

### Flow cytometry analysis

Control or CCDC86-depleted HeLa cells were subjected to cell cycle analysis by flow cytometry. Briefly, 1×10^6^ cells were fixed with ice-cold ethanol (70%) for 1 h, centrifuged and resuspended in PBS containing RNase A (0.2 mg/ml, Sigma-Aldrich) and propidium iodide (10 μg/ml; Thermo Fisher Scientific, P3566). Following a 20 min incubation, the cells were analysed by flow cytometry using a ACEA NovoCyte flow cytometer (Agilent). The FL2 channel (488 nm laser) was used to analyse 20,000 events per condition. Gated cells were manually categorised into cell cycle stages. The cells were analysed following knockdown periods of 48 and 56 h.

### Antibodies

The following primary antibodies were used for immunofluorescence: anti-Ki-67 (1:100, mouse monoclonal, BD Transduction laboratory, Oxford, UK, 610968); anti-nucleolin (1:300, rabbit polyclonal; Abcam, ab22758); anti-α-tubulin antibody (1:10,000, Sigma-Aldrich, B512), anti-GFP (1:1000, Invitrogen, A-11122); anti-lamin A/C (1:2500, Abcam, ab108595) and anti-GFP (1:2000, ChromoTek, PABG1-20). The anti-centromere antibody (1:5000) comes from the collection of W.C.E.

### Indirect immunofluorescence

For immunofluorescence, cells were fixed in 4% paraformaldehyde and processed as previously described (Vagnarelli et al., 2011). Fluorescently labelled secondary antibodies were applied at 1:200 (Jackson ImmunoResearch, 715-585-150 and 715-096-150 ). Three-dimensional data sets were acquired using a cooled CCD camera (CH350, Photometrics) on a wide-field microscope (DeltaVision Spectris; Applied Precision) with a NA 1.4 Plan Apochromat lens. The data sets were deconvolved with softWoRx software (Applied Precision). The three-dimensional data sets were then converted to Quick Projections in softWoRx. Three-dimensional data sets were also acquired using a wide-field microscope (Nikon Ti-E super research Live Cell imaging system) with a NA 1.45 Plan Apochromat lens. The data sets were deconvolved with NIS Elements AR analysis software (Nikon). The three-dimensional data sets were converted to Maximum Projections using the NIS software. In both cases, the images were exported as TIFF files, imported into Adobe Photoshop and then imported into Inkscape for final presentation.

#### Live-cell imaging

Live cell imaging for Fig. 2E was performed with a DeltaVision microscope as previously described (Vagnarelli et al., 2011).

For Fig. 4G,H and Fig. S2, HeLa cells were seeded onto Labtech chamber slides with complete Leibovitz's L-15 medium, lacking phenol red (Gibco). Cells were imaged by differential interference contrast microscopy with a 20× objective (NA 0.45) and a wide-field microscope (NIKON Ti-E super research Live Cell imaging system) at 37°C. Images were captured every 30 min over a period of 24.5 h. Analyses of mitotic progression were conducted manually.

### PLA

PLA was performed according to the manufacturer's protocol (Duolink PLA Control Kit, DUO92202-1KT, Sigma-Aldrich). HeLa Kyoto cells were transfected with plasmids encoding GFP–CDC86^wt^, GFP–CDC86^Δ63^ or GFP. At 24 h post transfection, the cells were fixed, permeabilised and blocked with bovine serum albumin as described above. The anti-Ki-67 and anti-GFP antibodies were used at the concentrations indicated in the section ‘Antibodies’. PLA probes were added and ligation was performed following the manufacturer's instructions. The coverslips were mounted, counterstained with DAPI and observed with a wide-field Nikon microscope. Spots lying within nuclear masks were counted for the three conditions, and the number of foci were used to generate the violin plots in R.

### Focus diameter quantification

HeLa Kyoto cells were transfected with control or CCDC86 siRNAs. At 48 h post transfection, the cells were fixed, permeabilised, blocked with bovine serum albumin and immunostained as described above. The antibodies were used at the concentrations indicated in the section ‘Antibodies’. The focus number was quantified on maximum-projection images. The diameter of the spots lying within nucleolin masks were counted in the control and CCDC86 siRNA using ImageJ and the numbers were used to generate the violin plots in R.

### qPCR

The total RNA of TET21-N cells (control and 24 h treatment with doxycycline) was extracted using the Monarch Total RNA Miniprep Kit (New England Biolabs, Hitchin, UK), from which complementary DNA (cDNA) was synthesised by reverse transcription using RevertAid RT Reverse Transcription Kit (Thermo Fisher Scientific). qPCR was performed using Maxima SYBR Green/ROX qPCR Master Mix (2×) (Thermo Fisher Scientific) using the QuantStudio 7 Flex Real-Time PCR Instrument (Thermo Fisher Scientific). The relative gene expression was calculated using the comparative Ct method (ΔΔCt). GAPDH was used as the endogenous reference gene.

qPCR was conducted using the following primers: GAPDH Fw, 5′-ACCACAGTCCATGCCATCAC-3′; GAPDH Rev, 5′-TCCACCACCCTGTTGCTGTA-3′; MYCN Fw, 5′-CACAAGGCCCTCAGTACCTC-3′; MYCN Rev, 5′-ACCACGTCGATTTCTTCCTC-3′; CCDC86 Fw, 5′-TTCCTCTCCTGTCGTTCCTT-3′; and CCDC86 Rev, 5′-AGCGAAAAGGTTCTTCATCC-3′.

### *In silico* neuroblastoma analyses

The analyses of Neuroblastoma patient data sets were conducted using the R2: Genomics Analysis and Visualisation Platform (http://r2.amc.nl). These data sets are available at GSE45547 and GSE49710.

### Statistical analyses

Statistical analyses were performed using a χ^2^ test, Fisher exact test or two-tailed unpaired Student's *t*-test.

## Supplementary Material

10.1242/joces.260391_sup1 Supplementary information
