# Supplementary material for: CCDC86 is a novel Ki-67-interacting protein important for cell division
Source: J Cell Sci. 2023 Jan 25;136(2):jcs260391. doi: 10.1242/jcs.260391 (PMC10022746; doi:10.1242/jcs.260391)
Supplement: Supplementary information [file joces-136-260391-s1.pdf]

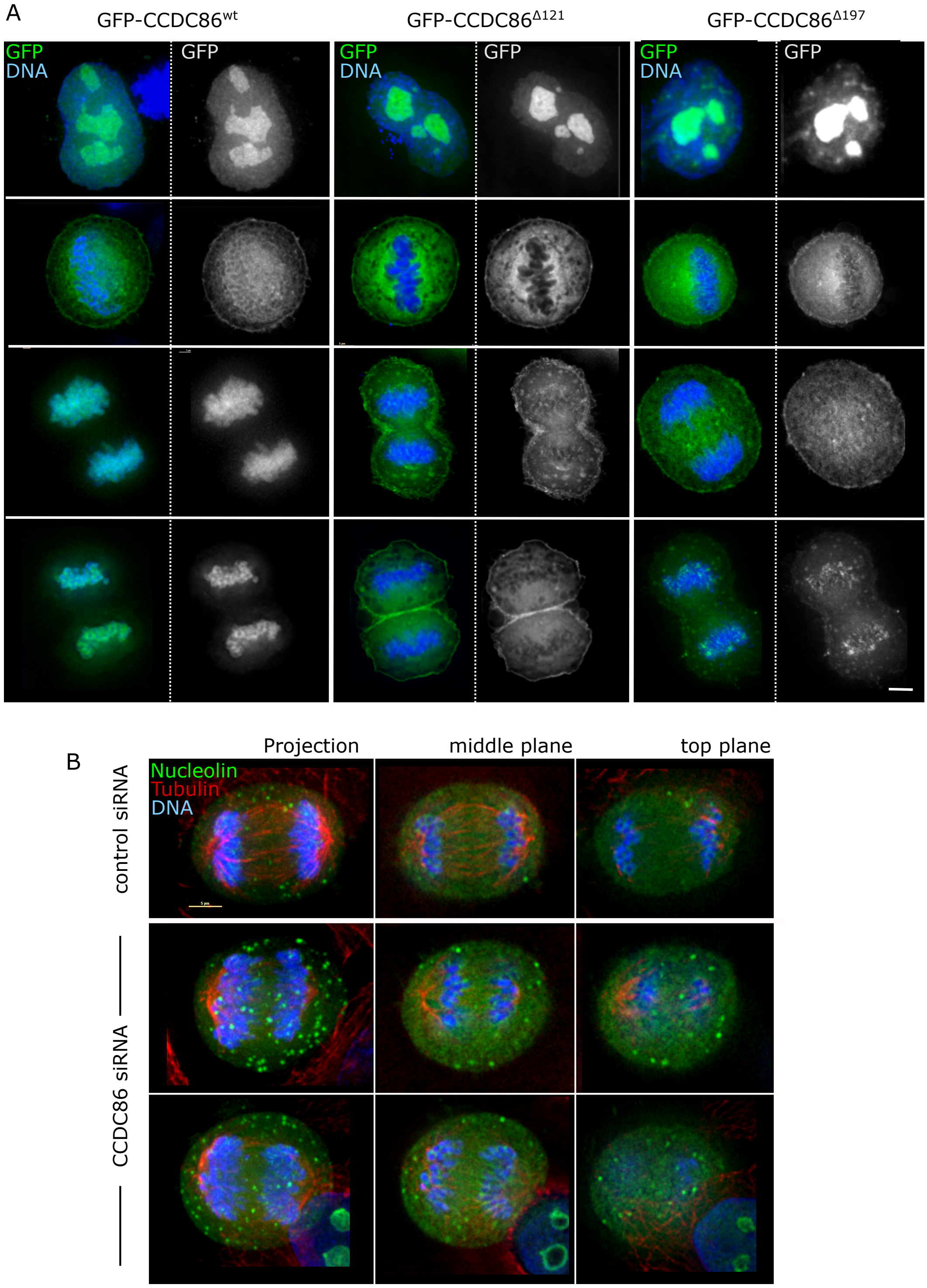

Fig. S1. A) Localisation of GFP-CCDC86^{wt}, GFP-CCDC86$^{\Delta 121}$and GFP-CCDC86$^{\Delta 197}$ in interphase (first row), prometaphase/metaphase (second row) and in anaphase/telophase (third and fourth rows). Scale bar 5 µm.

B) Localisation of the nucleolin foci in cells treated with control or CCDC86 siRNA relative to microtubules. For each cells Scale bar 5 µm.

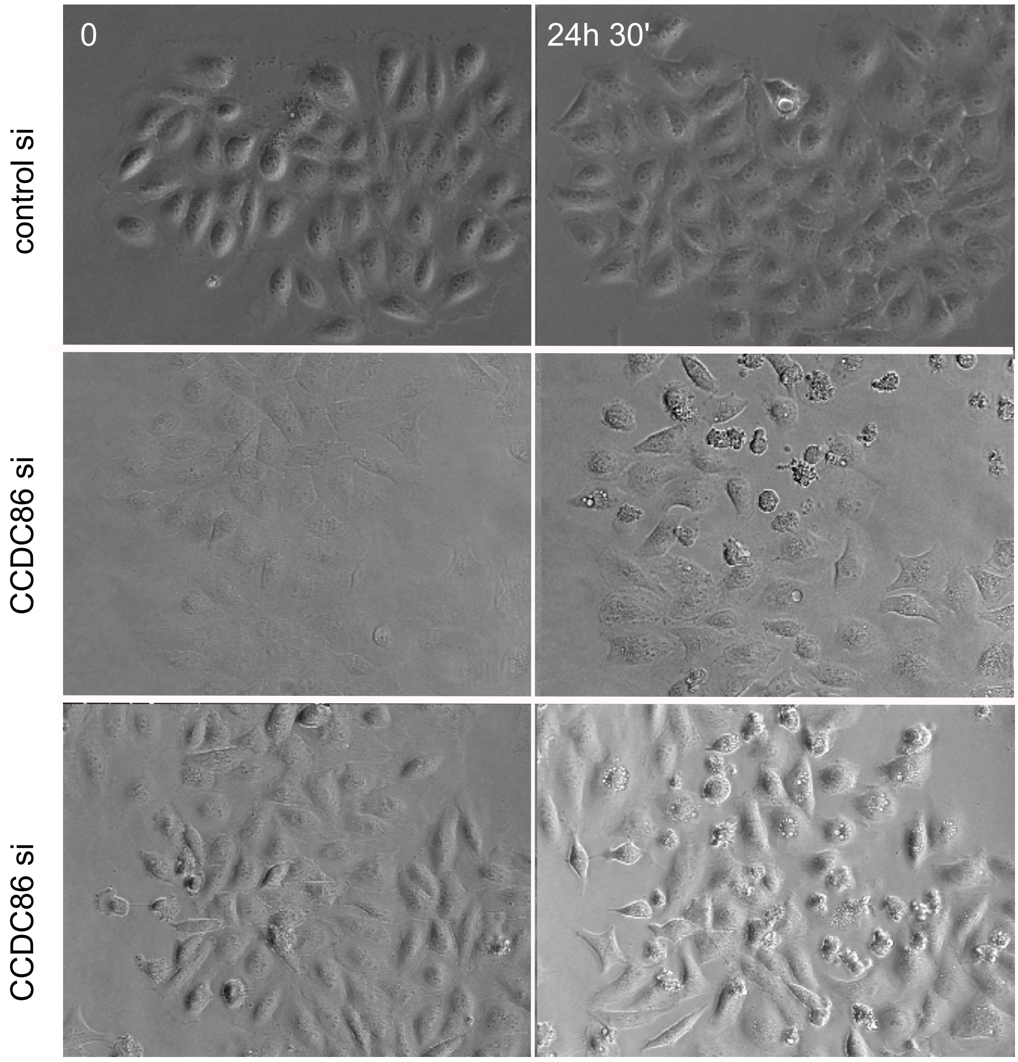

Fig. S2. HeLa cells were treated with control or CCDC86 siRNA and subjected to live cell imaging for 24.5 h. The figure shows representative images of a field of cells from both treatments at the beginning of the imaging and the same field at the end of the experiments.

Journal of Cell Science • Supplementary information
