# Supplementary material for: CCDC86 is a novel Ki-67-interacting protein important for cell division
Source: J Cell Sci. 2023 Jan 25;136(2):jcs260391. doi: 10.1242/jcs.260391 (PMC10022746; doi:10.1242/jcs.260391)
Supplement: Supplementary file 1 [file joces-136-260391_review_history.pdf]

CCDC86/Cyclon is a novel Ki-67 interacting protein important for cell division

Konstantinos Stamatiou, Aldona Chmielewska, Shinya Ohta, William Earnshaw and Paola Vagnarelli
DOI: 10.1242/jcs.260391

Editor: David Glover

Review timeline
Original submission: 4 July 2022
Editorial decision: 1 September 2022
First revision received: 31 October 2022
Accepted: 8 December 2022

Original submission

First decision letter

MS ID#: JOCES/2022/260391

MS TITLE: CCDC86/Cyclon is a novel Ki-67 interacting protein important for cell division

AUTHORS: Konstantinos Stamatiou, Aldona Chmielewska, Shinya Ohta, William Earnshaw, and Paola Vagnarelli
ARTICLE TYPE: Research Article

We have now reached a decision on the above manuscript.

To see the reviewers' reports and a copy of this decision letter, please go to: https://submit-jcs.biologists.org and click on the 'Manuscripts with Decisions' queue in the Author Area. (Corresponding author only has access to reviews.)

As you will see, the reviewers raise a number of substantial criticisms that prevent me from accepting the paper at this stage. They suggest, however, that a revised version might prove acceptable, if you can address their concerns. If you think that you can deal satisfactorily with the criticisms on revision, I would be pleased to see a revised manuscript. We would then return it to the reviewers.

Please ensure that you clearly highlight all changes made in the revised manuscript. Please avoid using 'Tracked changes' in Word files as these are lost in PDF conversion.

I should be grateful if you would also provide a point-by-point response detailing how you have dealt with the points raised by the reviewers in the 'Response to Reviewers' box. Please attend to all of the reviewers' comments. If you do not agree with any of their criticisms or suggestions please explain clearly why this is so.

Reviewer 1

Advance summary and potential significance to field

Stamatiou et al. present work highlighting the role of CCDC86/Cyclon in mitosis. This protein is part of the chromosome periphery, an enigmatic chromosomal compartment, and interacts with the

© 2022. Published by The Company of Biologists under the terms of the Creative Commons Attribution License (https://creativecommons.org/licenses/by/4.0/).

best characterised protein of this compartment, Ki67. There is plenty of interest in the characterisation of the chromosome periphery, and people working in this area are likely to want to read this work. The results seem pretty straightforward and support the main conclusions, but in our opinion the paper would be improved by further quantification.

Comments for the author

Fig 2A: the authors state that “Ki67 RNAi strongly diminished the enrichment of CCD86 at the periphery of the chromosomes both in early mitosis and during mitotic exit”. Looking at this figure, the coating is less around chromatin in prometaphase but not really in telophase. Some quantification of the two pools (chromosome associated and soluble/cytoplasmic) would help the authors' case.

The results in 2C and 2D are very interesting. Are the defects worse with all three AT-hooks deleted? It also seems like there is an effect on DNA condensation/compaction with Δ63 mutant. Is it possible to measure such an effect to see if that is the case?

Fig 3D: we found these results really interesting and some more detail on the phenoptype would be nice. What is the size of an average foci? The distribution of the NDFs in mitotic cells: is it uniform or are they excluded from spindle regions? Do NDFs take Ki67 from the coating or is it a new population?

Fig 4: it looks from the cytometry that there is a clear sub-G0, suggesting an apoptotic pathway. It would be good to know the fate of the mitotic cells, i.e. are they doing slippage and into apoptosis? It would be nice to see a fate diagram for cells with and without mitotic errors. This could be compared with Ki67 siRNA as a positive control of bad fate outcomes.

Minor

1. Scale bars: some panels have 3 in identical subpanels of one condition yet none in the other (1E) or are missing them (1D). If all subpanels are the same mag, 1 scale bar in the bottom right works well, and if not a bar for each condition or whatever is fine.

2. The term "GFP:CCDC86" is used throughout the paper. It is conventional for mammalian expression to use GFP-CCDC86 when GFP is fused at the N-terminus of CCDC86. The colon nomenclature is - I believe - reserved for when GFP expression is linked to the other gene but not by direct fusion. I would advise changing this to avoid confusion.

3. The stacked bar charts in 3C,F,4C,E - at least on the reviewer copy have a weird shading on the red fill that makes it look like there are subclassifications.

4. Survival plot in 5A has stretched text - replot

5. Typo in Spindle length (μm) in Fig 4F

6. Fig 5F test is too small to read.

7. Fig 2A has an extra row, which is a duplicate of the first but without DNA. Not sure what this adds.

8. NDF needs to be defined earlier.

9. p. 12 I think what is shown is flow cytometry not FACS?

<u>Reviewer 2</u>

Advance summary and potential significance to field

CCDC86 has been identified by the authors of the manuscript as a new component of the chromosome periphery network. The authors have made an attempt to define some features of this

© 2022. Published by The Company of Biologists under the terms of the Creative Commons Attribution License (https://creativecommons.org/licenses/by/4.0/).

protein, including localisation pattern in cells of the chosen cell line and the phenotype that these cells show after the CCDC86 depletion.

These findings add to the knowledge within the field of chromosome biology and contribute to the understanding of how this subset of proteins bound to mitotic chromosomes may affect the progression of mitosis and proper chromosome segregation. This is within the merits of Journal of Cell Science and the study potentially may be of interest of a broad audience of scientists working on cell division and the biochemistry of the mitotic apparatus.

Below I am suggesting the additional work that should be considered in order to improve the manuscript, so it meets important standards in the field. While I am leaving the decision about the acceptance of the manuscript entirely to the editor, I would like to suggest a revision of this manuscript before it could be considered for the publication in JCS.

Comments for the author

I find the study very interesting, however the manuscript in its current form does not seem to be ready for the publication.

There are three major areas, which I would like to focus on.

1. The manuscript is overall very "untidy". There are some typos, which I am not going to identify here, but instead I will mention the following issues: (1) in "Introduction" a paragraph that starts with "Four different functions (…)" is not properly referenced; please support each suggested function by a reference (at least one). (2) In "Methods" a subchapter "In silico (…)" is repeated. (3) Scale bars are missing on figure 1D, 2A and 2D. (3) One column (right hand side) and three rows of images shown in Fig 1D have no description (captions) on the figure. Please, describe the images properly. (4) Figure 2A: "CCDC86" should be replaced by either the full name of the construct (fusion protein) or "GFP"; in the current form it suggests that anti-CCDC86 antibody has been used. On the same panel, top row is "merge", the bottom row is also "merge". (5) I do not see Figure 3C ever mentioned in the text. (6) On page 13, "Figure 5C and D" should be corrected to "Figure 5C and E". (7) On page 16, the text "Interestingly, even though Ki-67 is necessary for CCDC86 recruitment to the chromosomes, it is not sufficient. A mutant version of GFP:CCDC86Δ63, lacking the first AT-Hook domain, abolished the localization of the protein to mitotic chromosomes." should be rephrased. This fragment is mixing up a necessity of the Ki-67 presence for the proper localisation of CCDC86 with the requirement for the CCDC86 protein to be intact. These are two independent features – a certain part of any protein is always needed for the localisation to a define place in the cell.

2. Data presentation. In Figure 1A, CCDC86 is shown in red on both graphs. Please, add at least one other protein from the chromosome periphery to illustrate how a different analysis of the previously acquired data may contribute to a better understanding of a group of proteins localising to the same compartment in the cell (different colours can be used, obviously). For panel 1C, please, add in the text an explanation as for how the visualisation of the network is made. Are the connections between proteins made from the data extracted from the publications showing predictions (potentially generated in silico) or "real" results from the protein-protein interaction studies (biochemistry)? In Figure 2A, please, comment on the pattern of the CCDC86-GFP fusion, which after a Ki-67 RNAi suddenly localises only to the nuclear envelope. A short comment in "Discussion" will be sufficient. Figure 3B: the authors made a decision about assessing an RNAi efficiency by using the GFP fusion to the target. Although this approach is not without merit, it would have been better to assess the knockdown in cells that do not express the fusion, by using the anti-CCDC86 antibody. Antibodies against CCDC86 are available (AtlasAntibodies) and their usage would also allow comparing the pattern of the GFP fusion localisation with the staining of the endogenous protein. Figure 3G: western shown here does not meet the quality standards. Unfortunately, a smear is present in a control lane at the level of the Ki-67, and this nonspecific signal does not allow the authors to state definitely whether there is any co-purification of Ki-67 or there is not. This western needs to be repeated. Moreover, I would like to suggest here adding a western after pull-down with the truncated form of CCDC86 (delta63) side by side with the full-length protein. The result may be insightful. It is important to show the good quality western, because at the moment it is the only result that supports the statement from the title about the

© 2022. Published by The Company of Biologists under the terms of the Creative Commons Attribution License (https://creativecommons.org/licenses/by/4.0/).

interaction with Ki-67. If this western is not repeated or does not show the interaction, the title needs to be modified.

3. Chromosome segregation. The authors studied the chromosome alignment phenotype that appears after the CCDC86 KD. The phenotype is quantified. Later in the discussion they make a connection between this phenotype and potential increased apoptosis rate (never confirmed by a specific, dedicated method). What is really missing here is the observation that addresses the question of the chromosome segregation during mitosis: do those misaligned chromosomes eventually end up segregated correctly, or not? And related to this, is the mitosis duration also affected? One simple way of addressing this question is filming cells after the RNAi and assessing the chromosome phenotype along with the timing of mitosis. This new part would significantly strengthen the chromosome phenotype observations and additionally would allow the authors to conclude something solid about this whole part of the phenotypical analysis.

First revision

Author response to reviewers' comments

We thank you very much for the time you have dedicated to overseeing the review of our manuscript.

We also thank both referees for the critique of the manuscript and the valuable comments and suggestions provided.

Taking into consideration their views, we have prepared a significantly revised version of the manuscript that address all the queries raised. We hope that our response will satisfy their requests and suggestions.

Below is a point-by-point reply to each comment where in blue are our answers and in green how the text has been modified in the new version of the manuscript.

Reviewer 1

We thank the referee for recognising the results as straightforward and supporting the main conclusions and for suggesting that the topic is of interest to the chromosome biology community.

The reviewer has raised several points that we have addressed as explained below:

1) Fig 2A: the authors state that “Ki67 RNAi strongly diminished the enrichment of CCD86 at the periphery of the chromosomes both in early mitosis and during mitotic exit”. Looking at this figure, the coating is less around chromatin in prometaphase but not really in telophase. Some quantification of the two pools (chromosome associated and soluble/cytoplasmic) would help the authors' case.
We have now quantified the recruitment of GFP-CCDC86 to the chromosomes in control and Ki-67 sRNA treated cells. The quantification shows that, upon Ki-67 depletion, GFP-CCDC86 cannot accumulate on the anaphase chromatin. The data are presented in the new Figure 2B.

2) The results in 2C and 2D are very interesting. Are the defects worse with all three AT-hooks deleted?
We cannot detect any difference during mitosis even when the 3 AT-hooks are deleted. We have added some images in Supplementary Figure 1 to document this statement. Now the text reads as follows:

“All these mutant fusions abolished the localisation of the protein to the chromosomes when analysed as transient transfections in HeLa cells (Figure 2 D and Supplementary Figure 1); The

© 2022. Published by The Company of Biologists under the terms of the Creative Commons Attribution License (https://creativecommons.org/licenses/by/4.0/).

localisation of the mutant in which all 3 AT-hook motifs are deleted shows the same localisation patterns as the GFP-CCDC86$^{\Delta 63}$ (Supplementary Figure 1 A)."

It also seems like there is an effect on DNA condensation/compaction with Δ63 mutant.
We have looked carefully at several images, but we did not see any obvious difference in chromosome compaction. We include below a series of images for the referee. If there is a subtile effect, this will require a more sophisticated method of analysis. The best would be to set up a dedicated FRET pair of Histones (such as the one developed by the Lamond lab) but this would be very laborious considering that a novel cell line and would need to be established, validated and a new FRET pair constructed as we have already GFP-tagged proteins). Because we do not observe any major differences at this point, the investment of time and resources to establish a new system is not a highest priority at this time.

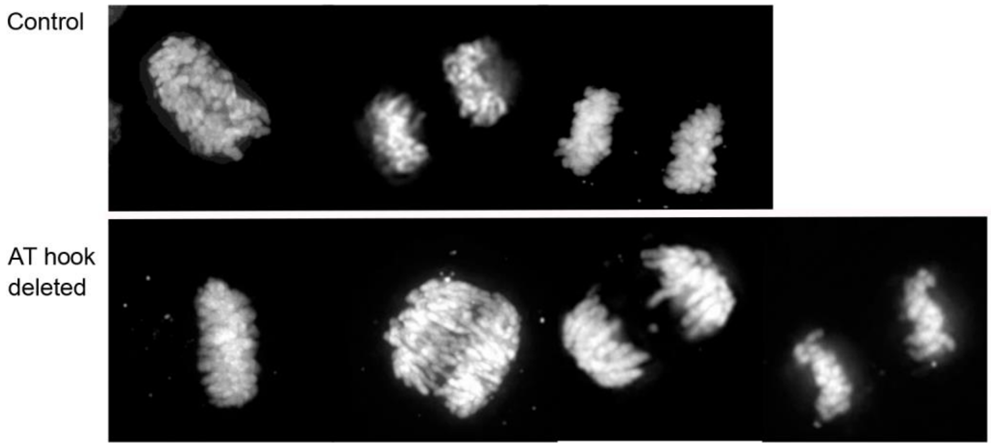

3) Fig 3D: we found these results really interesting and some more detail on the phenotype would be nice. What is the size of an average foci? The distribution of the NDFs in mitotic cells: is it uniform or are they excluded from spindle regions? Do NDFs take Ki67 from the coating or is it a new population?

We do agree that it is an interesting phenotype. As suggested by the referee, we have conducted further characterisation of the foci. We have quantified the number in anaphase/telophase cells (figure 3 E) and their diameter (Figure 3 F). We have also added some images to show that these foci are excluded from the spindle region in Supplementary Figure 1 B. The new text reads as follows:

Although Nucleolin is found in NDF foci in normal cells (Dundr et al., 2000), the foci were much bigger (Figure 3 F) and more abundant (Figure 3 E) after CCDC86 depletion and were also present in prometaphase/metaphase (Figure 3 C-F), which is not normal. Moreover, a larger number of foci are formed in CCDC86 depleted cells, and, interestingly, these are excluded from the spindle region (Supplementary Figure 1 B).
For the last question about Ki-67, although an interesting concept, it is quite a challenging investigation per se. In the future, we will pursue this interesting idea, but specific tools need to be built such as knock-in degron to endogenous CCDC86 to manipulate it at a specific time during cell cycle.
I hope the reviewer agrees that this is beyond the scope of this manuscript.

4) Fig 4: it looks from the cytometry that there is a clear sub-G0, suggesting an apoptotic pathway. It would be good to know the fate of the mitotic cells, i.e. are they doing slippage and into apoptosis? It would be nice to see a fate diagram for cells with and without mitotic errors. This could be compared with Ki67 siRNA as a positive control of bad fate outcomes.

We thank the referee for this suggestion. Yes, their interpretation is correct: cells without CCDC86 have an increase in apoptosis. As suggested, we conducted live cell imaging of HeLa cells upon control or CCDC86 RNAi and followed the population for 24h30'. The amount of apoptosis in the CCDC86 RNAi population is very high (we added some panels in Supplementary figure 2 A). We have

© 2022. Published by The Company of Biologists under the terms of the Creative Commons Attribution License (https://creativecommons.org/licenses/by/4.0/).

also analysed the progression and fate of mitotic cells. Now the data are summarised in Figure 4 F, G. We have cells that undergo apoptosis in mitosis but also cells that divide and then die after division. The percentage of cells that die upon division is strikingly similar to the percentage of cells showing alignment problems.
For Ki-67 RNAi, nobody has really provided evidence that there are mitotic errors. This represents a novel phenotype.
Now the revised text reads as follows.
Based on the flow cytometry data and the mitotic phenotypes, we then wanted to check if mitotic progression was altered in cells depleted of CCDC86 and the fate of cells after division. We therefore conducted live cell imaging of cells treated with control or CCDC86 siRNAs and imaged cells for 25 h every 30'. We recorded for all the cells that entered mitosis, the time each cell took from rounding up to cell elongation (anaphase/telophase), the duration between anaphase onset and the completion of division and the outcome of the daughter cells (if alive until the end of the experiment or the time they died after division).
The analysis of these experiments clearly shows that cells treated with control RNAi have an early mitosis length of 44' $\pm$ 18', a mitotic exit (anaphase-cytokinesis) of 30' and all survived after division until the end of the experiment. On the contrary, cells treated with CCDC86 siRNA, have an early mitosis length of 90' $\pm$ 282', a mitotic exit (anaphase-cytokinesis) of 67'$\pm$ 60' and, of the cells that entered mitosis, 12% died in mitosis and 17% died after division (either both daughter cells or one of them) (Figure 4 F, G). However, we noticed that some cells also died without dividing during the imaging time (compare the beginning and the end of the experiments in Supplementary Figure 2). At this point we do not know if those cells died because they divided before we started imaging or if because this protein, in addition to being required for a successful mitosis, has also some essential functions in interphase. Interestingly, the percentage of cells that died during or after mitosis (31 %) is very similar to the percentage of cells showing mitotic defects (figure 3 C)."

Minor

1. Scale bars: some panels have 3 in identical subpanels of one condition yet none in the other (1E) or are missing them (1D). If all subpanels are the same mag, 1 scale bar in the bottom right works well, and if not, a bar for each condition or whatever is fine.
We have fixed all the scale bars. Some of them seemed to have disappeared in the conversion of the files from svg to PDF. We have removed them for the panels that contained more than one.

2. The term "GFP:CCDC86" is used throughout the paper. It is conventional for mammalian expression to use GFP-CCDC86 when GFP is fused at the N-terminus of CCDC86. The colon nomenclature is - I believe - reserved for when GFP expression is linked to the other gene but not by direct fusion. I would advise changing this to avoid confusion.
We have replaced "GFP:CCDC86" with GFP-CCDC86.

3. The stacked bar charts in 3C,F,4C,E - at least on the reviewer copy have a weird shading on the red fill that makes it look like there are subclassifications.
We apologise. It was again created saving the .svg file to PDF.

4. Survival plot in 5A has stretched text – replot
We have fixed the problem.

5. Typo in Spindle length (µm) in Fig 4F
We have corrected the spelling.

6. Fig 5F test is too small to read.
We have increased the font size.

7. Fig 2A has an extra row, which is a duplicate of the first but without DNA. Not sure what this adds.
We have removed the panel.

8. NDF needs to be defined earlier.
We have defined the NDF earlier in the text.

© 2022. Published by The Company of Biologists under the terms of the Creative Commons Attribution License (https://creativecommons.org/licenses/by/4.0/).

9. p. 12 I think what is shown is flow cytometry not FACS?
The reviewer is correct. We have changed in the text.

Reviewer 2

We thank the referee for the useful comments and suggestions and for recognising not only that "*These findings add to the knowledge within the field of chromosome biology and contribute to the understanding of how this subset of proteins bound to mitotic chromosomes may affect the progression of mitosis and proper chromosome segregation*" but also that they "*may be of interest of a broad audience of scientists working on cell division and the biochemistry of the mitotic apparatus*."

The reviewer has raised several points that we have addressed as explained below:

Reviewer 2 Comments for the Author: I find the study very interesting, however the manuscript in its current form does not seem to be ready for the publication. There are three major areas, which I would like to focus on.

1. The manuscript is overall very "untidy". There are some typos, which I am not going to identify here, but instead I will mention the following issues:
We apologise for this and we have tried our utmost to make sure all those aspects have been taken care of.
(1) in "Introduction" a paragraph that starts with "Four different functions (…)" is not properly referenced; please support each suggested function by a reference (at least one).
We have added the reference beside each statement.

(2) In "Methods" a subchapter "In silico (…)" is repeated.
We have removed the repeated section

(3) Scale bars are missing on figure 1D, 2A and 2D.
We have added the scale bars. Some of them disappeared in saving the manuscript as .PDF from the .svg file.

(3) One column (right hand side) and three rows of images shown in Fig 1D have no description (captions) on the figure.
We have removed one column and we have added the mitotic stages for each row.

(4) Figure 2A: "CCDC86" should be replaced by either the full name of the construct (fusion protein) or "GFP";
We have changed it to GFP-CCDC86.

On the same panel, top row is "merge", the bottom row is also "merge".
The bottom row has been removed

(5) I do not see Figure 3C ever mentioned in the text.
Now it is mentioned in the text.

(6) On page 13, "Figure 5C and D" should be corrected to "Figure 5C and E".
We have corrected this.

(7) On page 16, the text "Interestingly, even though Ki-67 is necessary for CCDC86 recruitment to the chromosomes, it is not sufficient. A mutant version of GFP:CCDC86Δ63, lacking the first AT-Hook domain, abolished the localization of the protein to mitotic chromosomes." should be rephrased. This fragment is mixing up a necessity of the Ki-67 presence for the proper localisation of CCDC86 with the requirement for the CCDC86 protein to be intact. These are two independent features – a certain part of any protein is always needed for the localisation to a define place in the cell.
The sentence has been modified also taken into account the new PLA data.
The new text now reads as follows:

© 2022. Published by The Company of Biologists under the terms of the Creative Commons Attribution License (https://creativecommons.org/licenses/by/4.0/).

"Interestingly, a mutant version of GFP-CCDC86$^{\Delta63}$ lacking the first AT-Hook domain also failed to localize to mitotic chromosomes even though this mutant protein can still interact with Ki-67 *in vivo* (at least as assessed by PLA). This observation could indicate that CCDC86 has a chromosome periphery targeting module that does not solely depend on Ki-67 interaction. In interphase, upon Ki-67 RNAi, GFP-CCDC86 is diffuse in the nuclear space with enrichment at the periphery of the nucleoli, suggesting that its nuclear foci accumulation is also Ki-67 dependent."
"

2. Data presentation. In Figure 1A, CCDC86 is shown in red on both graphs. Please, add at least one other protein from the chromosome periphery to illustrate how a different analysis of the previously acquired data may contribute to a better understanding of a group of proteins localising to the same compartment in the cell (different colours can be used, obviously).

We have now visualised on the graphs a few other well know chromosome periphery proteins (with different colors) and modified the text accordingly as follows:
'…chromosomes from cytosol during the incubation as shown for Ki-67, cPERP-A - F and c12orf31, all chromosome periphery proteins (Ohta et al., 2011) (Figure 1 A, B)."

For panel 1C, please, add in the text an explanation as for how the visualisation of the network is made. Are the connections between proteins made from the data extracted from the publications showing predictions (potentially generated in silico) or "real" results from the protein-protein interaction studies (biochemistry)?
We have specified the source and how the database retrieves interactions in the text:
"GPS-Prot integrates human protein interactions derived from publicly available databases including MINT and BioGRID."

In Figure 2A, please, comment on the pattern of the CCDC86-GFP fusion, which after a Ki-67 RNAi suddenly localises only to the nuclear envelope. A short comment in "Discussion" will be sufficient.
The accumulation of CCDC86-GFP upon Ki-67 RNAi is not at the nuclear envelope. The signal is at the nucleolar periphery (Figure 2 A). We have added a sentence in the discussion and the text reads as follows:
"In interphase, upon Ki-67 RNAi, GFP-CCDC86 is diffuse in the nuclear space with enrichment at the periphery of the nucleoli, suggesting that its nuclear foci accumulation is also Ki-67 dependent."

Figure 3B: the authors made a decision about assessing an RNAi efficiency by using the GFP fusion to the target. Although this approach is not without merit, it would have been better to assess the knockdown in cells that do not express the fusion, by using the anti-CCDC86 antibody. Antibodies against CCDC86 are available (AtlasAntibodies) and their usage would also allow comparing the pattern of the GFP fusion localisation with the staining of the endogenous protein.

We have tried several commercial antibodies against this protein but, unfortunately, none of them have been validated. To our disappointment, either they do not recognise anything on blots, or they recognise too many things, none of them disappearing upon RNAi. This strongly suggests that all these antibodies do not recognise what they are reported to. I think, as a community, we need to be extremely careful in using commercial antibodies without strict validation.
Of course, it could be that there are some differences but, for most many studies there is a good overlap between the antibody and the GFP tagged proteins. However, the reverse could be also true, such as that the antibody only detects a subset of the protein, especially if the protein is subject to post-translational modifications or the epitope is masked by strong interactors.

Figure 3G: western shown here does not meet the quality standards. Unfortunately, a smear is present in a control lane at the level of the Ki-67, and this nonspecific signal does not allow the authors to state definitely whether there is any co-purification of Ki-67 or there is not. This western needs to be repeated. Moreover, I would like to suggest here adding a western after pull-down with the truncated form of CCDC86 (delta63) side by side with the full-length protein. The result may be insightful. It is important to show the good quality western, because at the moment it is the only result that supports the statement from the title about the interaction with Ki-67. If this western is not repeated or does not show the interaction, the title needs to be modified.
This was a good suggestion from the reviewer. CCDC86 has already been shown to co-IP with Ki-67 in Sobeki et al, therefore this was not a novel contribution. To go beyond this, we decided to take a

© 2022. Published by The Company of Biologists under the terms of the Creative Commons Attribution License (https://creativecommons.org/licenses/by/4.0/).

different approach and we conducted Proximity Ligation Assays (PLA) between Ki-67 and CCDc86 wt and, as suggested by the referee, the mutant form. This also provides more specific information on the site(s) where the interactions occur within the nuclear space. We have added these experiments in Figure 3 I, J. The new data show that both the wt form and the AT hook deleted version interact with Ki-67 all over the nucleus but also that there is some accumulation at the periphery of the nucleoli.
The modified text now reads as follow:
CCDC86 has been identified as a Ki-67 interactor upon Ki-67 pull-down followed by Mass spectrometry (Sobecki et al, 2016). Therefore, we wanted to investigate if the reported interactions between Ki-67 and CCDC86 could also be confirmed in cells and if the interaction was confined to a specific subcellular compartment. To look for association between Ki-67 and CCDC86 in vivo, we used a Proximity Ligation Assay approach (PLA). We used asynchronous HeLa cells transfected with either GFP or GFP-CCDC86^{wt} or GFP-CCDC86$^{\Delta 63}$ and conducted PLA experiments using anti-GFP and anti Ki-67 antibodies. The analyses show that numerous PLA signals can be obtained between Ki-67 and both versions of GFP-CCDC86 but not with Ki-67 and GFP alone (Figure 3 I, J). These signals are distributed throughout the nuclear space, but some enrichment can be detected at the periphery of the nucleoli (Figure 3 J).
We therefore conclude that CCDC86 is likely to interact with Ki-67 *in vivo* within the nucleus with some enrichment at the nucleolar periphery, and that the first AT hook domain of CCDC86 is apparently not essential for this interaction. However, because we did not conduct the PLA experiments in a CCDC86 knock down background, we cannot exclude that a dimer or a higher order complex including also the endogenous wt protein could yield a positive PLA result.

3. Chromosome segregation. The authors studied the chromosome alignment phenotype that appears after the CCDC86 KD. The phenotype is quantified. Later in the discussion they make a connection between this phenotype and potential increased apoptosis rate (never confirmed by a specific, dedicated method).
What is really missing here is the observation that addresses the question of the chromosome segregation during mitosis: do those misaligned chromosomes eventually end up segregated correctly, or not? And related to this, is the mitosis duration also affected? One simple way of addressing this question is filming cells after the RNAi and assessing the chromosome phenotype along with the timing of mitosis. This new part would significantly strengthen the chromosome phenotype observations and additionally would allow the authors to conclude something solid about this whole part of the phenotypical analysis.

This comment was also raised by referee 1 and we do agree it is important. We therefore did the requested live-cell analysis. While we do see increased mis-segregation of chromosomes upon CCDC86 RNAi, we did not have information on the timing of mitosis and the link to cell death.
Now these analyses obtained by live cell imaging are present in Figure 4 F, G.
Please see the response to Referee 1 - Point 4- for details.

<u>Second decision letter</u>

MS ID#: JOCES/2022/260391

MS TITLE: CCDC86/Cyclon is a novel Ki-67 interacting protein important for cell division

AUTHORS: Konstantinos Stamatiou, Aldona Chmielewska, Shinya Ohta, William Earnshaw, and Paola Vagnarelli
ARTICLE TYPE: Research Article

I am happy to tell you that your manuscript has been accepted for publication in Journal of Cell Science, pending standard ethics checks.

© 2022. Published by The Company of Biologists under the terms of the Creative Commons Attribution License (https://creativecommons.org/licenses/by/4.0/).

Reviewer 1

Advance summary and potential significance to field

I am happy with the authors' revisions to the paper, and their comments in response to my review.

Comments for the author

The authors have added new experimental data which addressed the main points I raised. They have provided good reasons not to investigate two points which were not crucial to the main point of the paper.

Reviewer 2

Advance summary and potential significance to field

The revised version is significantly improved.

Comments for the author

I believe that the authors have responded to all and fulfilled most of my earlier concerns. If the Editor agrees with this statement, and does not find any other reason to reject the current version of the manuscript, I would like to recommend acceptance for the publication.

Just one last point that I would like to make is that PLA is not a good substitute for western blotting, although - obviously - I can see some advantages of this change of approach.

The citation the is added in that new paragraph, which includes information about the earlier finding that the two proteins co-purify, (Sobecki et al, 2016), has NOT been added to the reference list. Please, correct this.

© 2022. Published by The Company of Biologists under the terms of the Creative Commons Attribution License (https://creativecommons.org/licenses/by/4.0/).
